# Process-Driven Optimization of FDM Porous PEEK Scaffolds for Alloplastic Bone Grafts

**DOI:** 10.1021/acsomega.5c06631

**Published:** 2025-09-30

**Authors:** Martina Galea Mifsud, Lucy Di-Silvio, Trevor Coward

**Affiliations:** Faculty of Dentistry, Oral & Craniofacial Sciences, 4616 King’s College London, London SE1 9RT, U.K.

## Abstract

Polyether ether ketone (PEEK) has emerged as a high-performance biomaterial for orthopedic and craniofacial applications due to its exceptional mechanical properties, chemical stability, and biocompatibility. Despite its clinical potential, the additive manufacturing of PEEK, particularly through fused deposition modeling (FDM), remains a considerable technical challenge owing to the polymer’s high melting point and narrow processing window. In this study, we report a novel and practical strategy for producing porous PEEK scaffolds with an optimized architecture suitable for bone graft applications. All initial CAD-based lattice designs failed under FDM processing conditions, consistently resulting in misprints with poor fidelity and structural inconsistencies. To address this, a process-driven approach through the adjustment of slicing parameters was adapted. Through iterative optimization, a reproducible scaffold design was achieved, with interconnected porosity and pore dimensions ranging from 100 to 400 μm, within the ideal range to support osteoblast adhesion, proliferation, and vascularization. The resulting scaffolds exhibited consistent morphology, mechanical integrity, and geometric fidelity, showing the importance of the slicing software parameters when used to circumvent computer-aided design limitations. This work demonstrates the pivotal role of manipulating the slicing software to unlock the full potential of high-performance thermoplastics such as PEEK in bone tissue engineering. Our findings offer a scalable pathway for producing customized, load-sharing scaffolds and open new avenues for integrating advanced manufacturing strategies in regenerative medicine.

## Graphical abstract

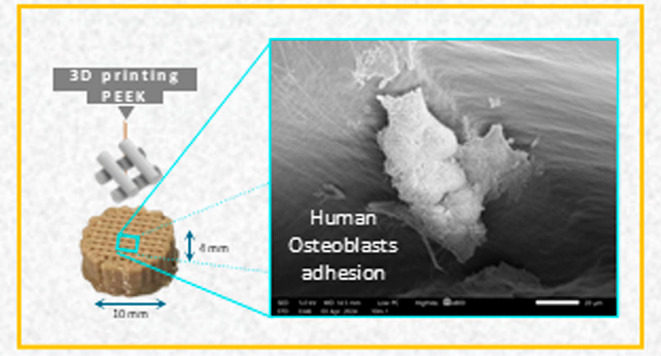

## 1 Introduction

### 1.1 The Need for Bone Grafts

The rising prevalence of bone pathologies driven by the increased human lifespan has amplified the demand for effective bone repair strategies. While autologous bone grafting remains the current gold standard, its use is limited by donor site morbidity associated with additional surgical intervention.^1^ Alloplastic, or synthetic bone grafts represent a promising alternative, particularly with advancements in 3D printing which enable the rapid fabrication of customized, complex structures through efficient design-manufacturing workflows. This technology makes it possible to produce patient-specific porous scaffolds precisely tailored to the geometric requirements of critical-sized bone defects.

An ideal porous scaffold designed for bone replacement should have mechanical stability^2^ and allow space for cell ingrowth^3^ while taking into consideration the fact that high porosity will negatively influence the material’s mechanical properties.^4^ Polyetheretherketone (PEEK), with its easy sterilization, low water absorption, and inherent biocompatibility, offers a material platform well-suited to address these competing demands, making it a preferred choice for orthopedic scaffolds.^5,6^

### 1.2 The Properties of PEEK

PEEK exhibits favorable mechanical properties, including high strength, wear resistance, and an elastic modulus comparable to that of bone,^7^ making it an ideal candidate for load-bearing and load-sharing implants, and bone replacement grafts. PEEK also exhibits excellent chemical stability and radiolucency,^7^ further enhancing its clinical applicability. However, despite these advantages, its intrinsic bioactivity remains limited,^8^ necessitating surface modifications or composite approaches to enhance cellular response, osseointegration, and overall biocompatibility. Addressing these challenges is critical for optimizing PEEK’s performance in bone tissue engineering and ensuring its viability as a long-term grafting material.

Of significant interest in orthopedic applications due to, among others, its elastic modulus, PEEK offers improved integration and mechanical compatibility for load-bearing and load sharing applications. Implant load-bearing and load-sharing requirements vary by application, and PEEK can be engineered with mechanical properties tailored to meet the demands of different orthopedic and spinal devices.^7^ Neat PEEK has a Young’s Modulus (YM) of 3.6 GPa, with its carbon fiber-reinforced version having an 18 GPa YM.^9^ This is well within the parameters of bone, whose YM is 10.4 to 14.8 GPa for the trabecular bone and 18.6 to 20.7 GPa for the cortical bone.^10^ These values give rise to the desired load sharing rather than stress shielding, which can be a drawback of titanium when used for orthopedic purposes due to its relatively high YM.^11^ PEEK is also radiolucent, allowing clear imaging of underlying tissues during body scans^12^ (Figure 1).

**Figure 1:**
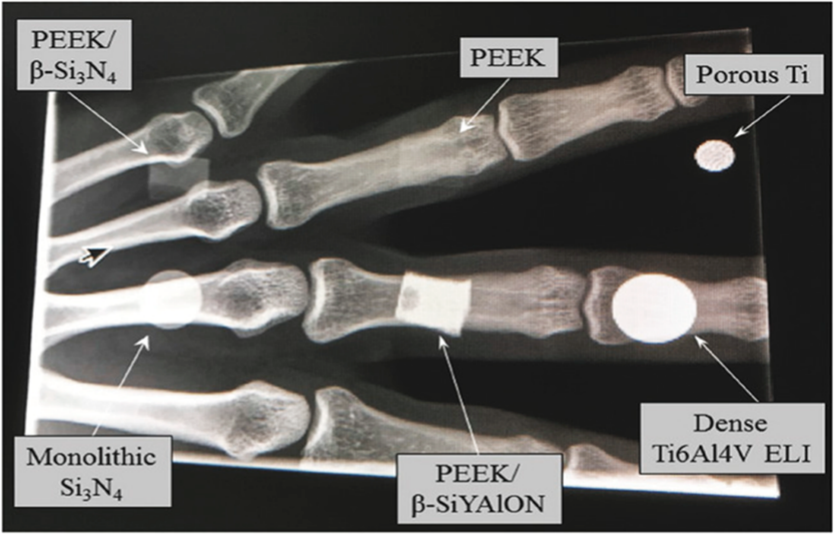
“X-ray radiograph comparing the translucency performance of three composites in comparison with the monolithic PEEK control and other biomaterials employed as orthopedic implants”. Reproduced with permission from Pezzotti et al. (2018) Macromolecular Bioscience. Copyright 2018, John Wiley and Sons.^13^

PEEK (C_19_H_14_O_3_) is a chemically inert thermoplastic, eliciting no cytotoxicity or mutagenicity *in vitro*.^14^ It has low water solubility at 0.5 w/w %^7^ and an adequately high glass transition temperature *T*_g_ of 145 °C.^15^ These characteristics make the material chemically adequate for use in the human body. PEEK is also corrosion-resistant, which is crucial for a biomaterial inside the harshly corrosive environment of the human body. This is another advantage over metal materials, for whom “chemical corrosion is inevitable”.^16^

Despite its favorable physical and chemical properties, PEEK is often overlooked as a bone-grafting substitute due to its limited bioactivity and ability to integrate, despite FDA recognition as a biocompatible implant material.^17^ Interestingly, the literature presents conflicting findings regarding the bioactivity of PEEK when compared to titanium, which is a well-known implant material.^18^ Some studies report no significant differences in osteoblast proliferation between the two materials,^6,19^ while others highlight a clear lack of similarity in their bioactive properties.^11,20^ Despite these discrepancies, a consistent concern across studies is PEEK’s insufficient osseointegration, which remains a primary limitation of its clinical application.^8^

### 1.3 Improving PEEK’s Bioactivity

Several methods have been described to improve the bioactivity of PEEK, essentially encompassing two main approaches: Composite Fabrication and Surface Modification^5^ (Figure 2), as this would interface with the host tissues and determine its ability to osseointegrate. Numerous studies have aimed to improve PEEK’s performance through interventions such as tertiary surface coatings and structural modifications, including composite fabrication (Table 1). Alternatively, the introduction of porosity within the PEEK structure will also improve its biological responsiveness.^5^

**Figure 2:**
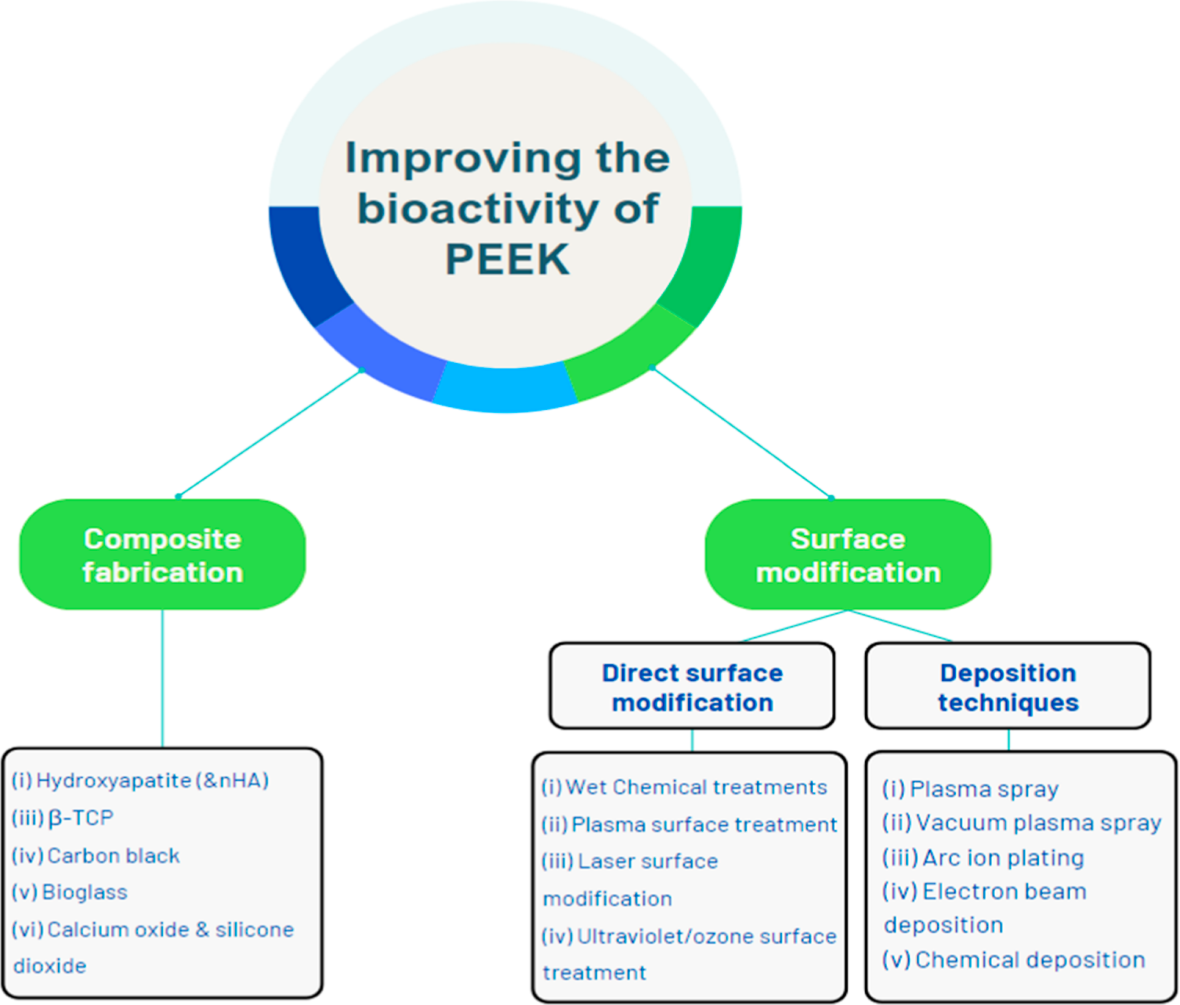
Overview of various strategies to enhance the bioactivity of PEEK. Approaches include structural alterations like composite fabrication and surface modifications such as physical or chemical coatings. These methods aim to improve cell adhesion, proliferation, and overall biological integration of PEEK-based implants.

**Table 1:**
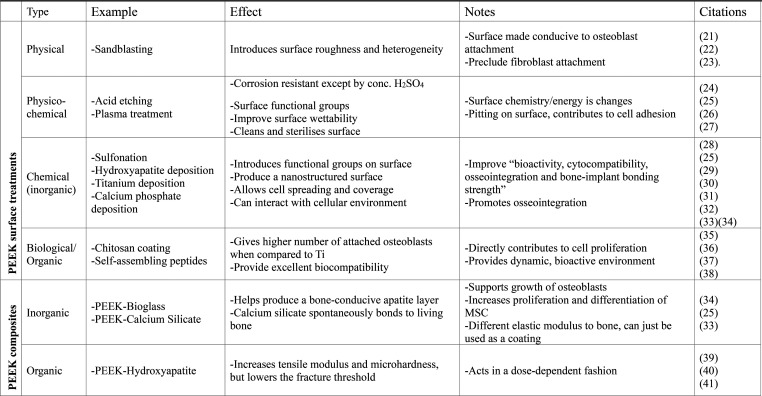
Comparing PEEK Interventions to Improve Its Bioactivity^21,22,23,24,25,26,27,28,29,30,31,32,33,34,35,36,37,38,39,40,41^

### 1.4 Manufacturing of PEEK

There are two main ways of manufacturing PEEK in industry: either through additive or subtractive manufacturing, i.e., 3D printing/SLS and milling. Given that orthopedic porous materials require interconnected pores of appropriate size to be able to support biological function, the fabrication of PEEK-based scaffolds has been explored; however, optimizing this process remains challenging.^42,43^ To circumvent these challenges, current methods of industrial fabrication have been transposed to the medical industry, such as sintering, micromachining, and particulate leaching,^8^ but these are still limited in terms of what can be produced. Of notable superiority over the other methods, extrusion free-forming has been recognized as the most efficient way of manufacturing porous 3D scaffolds due to its reproducibility, controllability, and customizability.^44^ These scaffolds can vary in internal geometries, with the diamond internal structure showing the highest interconnectivity and thus higher permeability characteristics.^45^ A similar porous scaffold was attempted in this research. In scaffold design for bone grafting, pore dimensions are key determinants of cell adhesion, proliferation, and eventual differentiation. In the available literature, there is no consensus about the optimal pore size for the ideal orthopedic scaffold, but the very minimum is recommended to be around 100 μm,^46^ while the maximum should be over 300 μm,^47^ even up to 600 μm^48^ to allow adequate vascularization.

The 3D printing of PEEK is complex and not easily achieved. Its relatively high melting temperature and elevated viscosity,^49^ have slowed progress in the development in additive manufacturing (AM) with this material. Moreover, PEEK is inherently prone to incomplete crystallization upon cooling, as well as distortion, both of which compromise the final mechanical properties of the printed construct.^50^ To address these challenges, newer printer models employ heated printing chambers to regulate crystallisation during cooling and thereby minimise distortion.^51^ In the printing of this construct, a higher printing speed was employed to prevent the collapse of internal struts by ensuring rapid cooling and preservation of the intended geometry.

## 2 Materials and Methods

### 2.1 Fabricating a Solid Disc

Initially, the study focused on the fabrication and optimization of a porous scaffold of PEEK using an Apium P155 filament printer (Apium Additive Technologies GmbH, Germany). Since PEEK is notoriously challenging to additively manufacture (Section 1.4), the printing parameters within the slicing software had to be optimized to achieve the desired outcome. The process required precise control of a number of software parameters ultimately affecting pore and channel dimensions within a defined range while also ensuring an interconnected architecture conducive to subsequent cell migration.

Initially, this printer was used to fabricate discs of 10 mm (diameter) × 4 mm (height) with defined parameters (Table 2):

**Table 2:** Original Disc Parameters, Used to Fabricate Solid Discs of 10 mm by 4 mm

Parameter	Value
Top Layers	3
Bottom Layers	3
Side/Perimeter Layers	3
Infill Pattern	Grid
Infill Angle Offsets	45°
Infill Percentage	20%

### 2.2 Fabricating a Scaffold—Optimization

A predetermined combination of printing parameters was used as a foundation for this project. The parameters (Table 2) had been used prior (by the author) to fabricate a 10 mm by 4 mm solid disc of PEEK, which had been carried out successfully. The computer-aided design (CAD) was subsequently modified in Autodesk Inventor (version 29.0), changing the construct from a solid cylinder to a porous geometry (initially 150 μm pores) before slicing for printing. The original slicing parameters were applied, however although these had successfully produced the original solid discs,they proved ineffective for the porous scaffold, making further optimization of the printing conditions necessary. The slicing software parameters were therefore adjusted accordingly, varying every single one initially in isolation and subsequently in combination, retaining the original solid CAD model rather than the porous CAD model. Among the changes applied were the following, with the respective values before and after optimization (Table 3).

**Table 3:** Printing Parameters Adjusted, with the Values Found before and the Improved Values after^a^

	Parameter	Value Before	Value/s Changed
Layer	Top Layers	3	0
	Bottom Layers	3	0
	Side/Perimeter layers	3	0
			
Infill	Infill Pattern	Grid	Honeycomb (HC), Rectilinear (RL), Wiggle
	Infill Angle Offsets	45°	45°
	Infill Percentage	20%	50%, 60%, 85%, 100%
			
Additions	Outline Overlap	90%	10%, 50%
	Skirt/Brim Outlines	10	20, 30
	Skirt Offset from Part	4.20	0
			
Temperature	Printing Temperature	486 °C	520 °C, 450 °C, 435 °C
	Heated Bed Temperature	150 °C	130 °C
	Cooling Layer 1	0%	0%, 60%, 100%
			
Other	Extrusion Multiplier	0.9	0.85, 0.08
	Printing Speed	4800 mm/min	2400, 1200, 800 mm/min
	Generate Support Material	√	X
	Internal Thin Wall Type	Perimeters Only	Allow Single Extrusion Fill

a The software tab headings for each of these parameters are found in the leftmost column.

Through iterative optimization, the parameters were determined and used for all subsequent discs (Table 4).

**Table 4:** Key Parameters which Created the Scaffold^a^

Parameter	Optimized Value
Infill Pattern	Rectilinear
Infill Angle Offsets	Alternating 45°, –45°
Infill Percentage	50%

a Using these parameters, the software created the scaffold through adjustment of the slicing settings.

### 2.3 Preliminary Biological Work

To determine the biological potential of the fabricated scaffold, a primary human osteoblast cell (HOB) model (C-12720, PromoCell, Heidelberg, Germany), with cells isolated from femoral trabecular bone tissue, was set up. These were initially cultured in a 75 cm^2^ cell culture flask with the standard culture growth medium containing DMEM (D6046 Sigma-Aldrich USA) supplemented with 10% Fetal Bovine Serum (F9665 Sigma-Aldrich, USA) as well as 1% Penicillin–Streptomycin (P0781 Sigma-Aldrich, USA). The HOBs were cultured at 37 °C in a 95% humid, 5% CO_2_ atmosphere. The complete medium was changed every 3 days. After confluence, the HOBs were detached using trypsin (T4174 Sigma-Aldrich, USA) and quantified using Trypan Blue (T8154 Sigma-Aldrich, USA) and a cell counter (TC10­(TM) Automated Cell Counter, Bio-Rad, USA). 4 × 10^5^ cells were microseeded on each scaffold within a 48-well plate. The scaffolds were left to incubate for 30 min, after which the complete medium was added. This cell model was cultured over a period of 21 days, with medium changes every 3 days taking care to not disrupt the flow of the medium to minimize forces on any cells attached. The scaffolds were then fractured, gold sputtered, and imaged using SEM (JCM-7000 NeoScope (TM), JEOL, Japan).

## 3 Results and Discussion

The main aim was to print and optimize a PEEK scaffold, using FDM printer Apium P155. This printer was used to fabricate discs with designed dimensions 10 mm (diameter) by 4 mm (height) using the parameters in Table 4, which would be optimal for use in a 48-well tissue culture plate for their biological assessment in due course. The optimization process yielded a range of results that informed each subsequent stage. Initial construct parameters were based on preliminary work carried out by the same author,^52^ employing a cylindrical shape with fixed parameters, optimized for a solid surface to support cell seeding. In that context, the internal structure and infill density were secondary considerations, with the 20% infill density arbitrarily selected to balance structural integrity and material efficiency. Once the solid-surface discs were successfully printed, the first porous scaffold was designed with homogeneously distributed 150 μm pores in an Autodesk Inventor (Figure 3).

**Figure 3:**
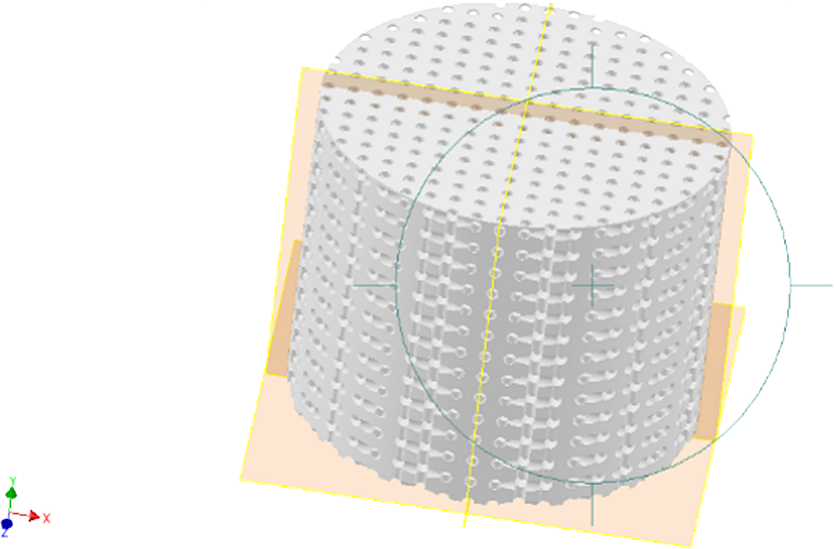
Design of the scaffold with 150 μm pores in a rectangular pattern. Designed using Autodesk Inventor (R), (version 29.0) Reproduced with permission. Licensed for educational purposes only.

When the sample was processed through Simplify3D­(R) slicing software with the same printing parameters, the print failed (Figure 4). Notably, while the software includes over 80 parameters, the most influential ones were those discussed here (Table 4).

**Figure 4:**
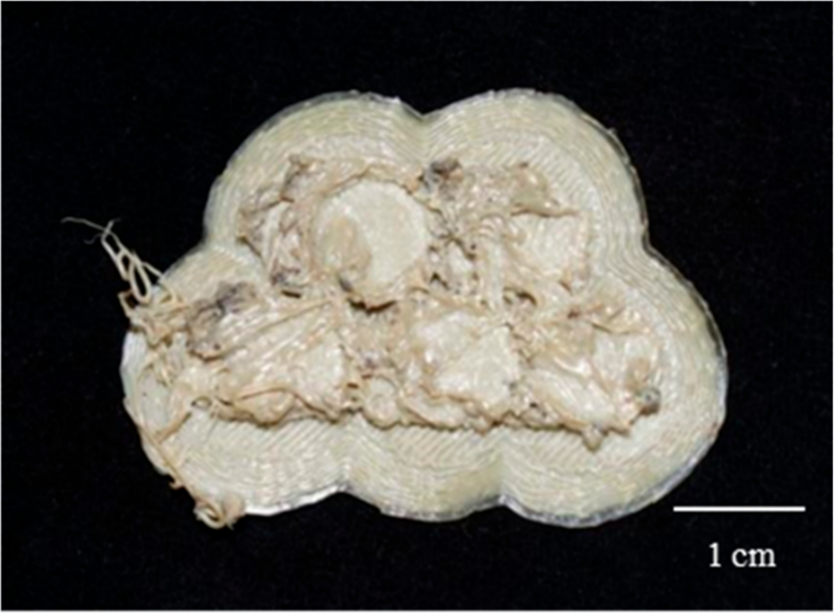
First attempt of 10 mm by 4 mm discs with 150 μm pores distributed throughout. Printed using Apium P155.

The designed scaffold was printed multiple times. Initially, the design remained the same, with 150 μm pores homogeneously distributed throughout the 10 mm × 4 mm construct. To improve the outcome, the slicing parameters were adjusted in an iterative manner to optimize the construction of the design. Parameters modified included nozzle temperatures, bed temperatures, and printing speed; however, misprints still occurred. A cube was eventually tested instead of a cylinder disc under the assumption that the printer was struggling with the circular geometry. However, due to the relatively low nozzle temperature at the time, the cube delaminated and separated into two halves upon completion. The interior of the printed construct revealed insights into the printing process (Figure 5).

**Figure 5:**
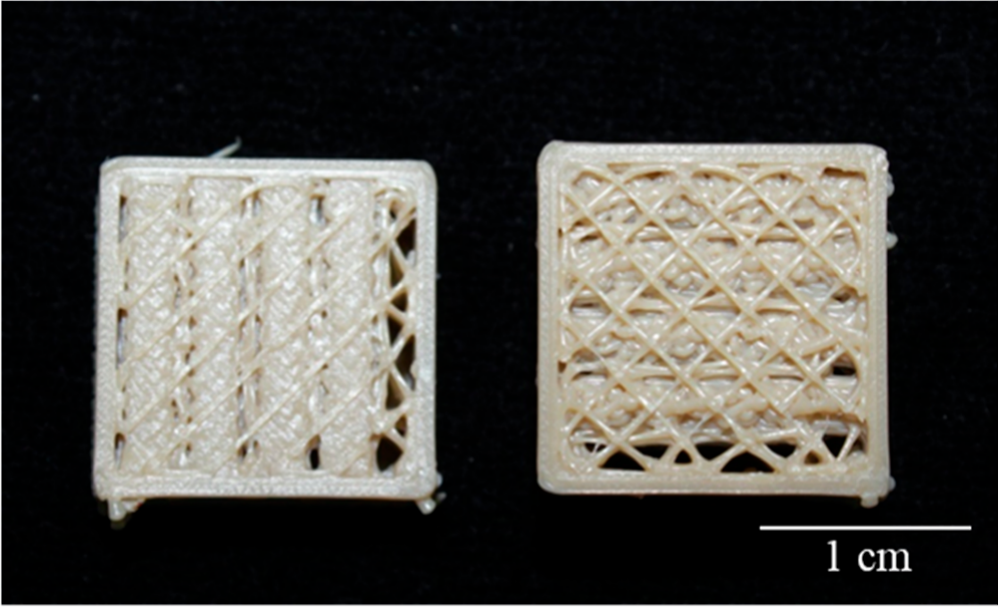
A delaminated AM cube, showing the internal geometry.

It became evident that the printer was attempting to fabricate the channels while simultaneously trying to execute the infill pattern specified in the slicing software. Given the failure of previous attempts to print the designed disc with porous inclusions, a decision was made to manipulate the slicing software to achieve the desired pore structure. The CAD design was reverted to a solid disk throughout, with the intention of manipulating the slicing software instead of the design software to fabricate the porous scaffold.

For most of the parameters, adjustment in isolation did not significantly affect the overall outcome. A variety of parameters were adjusted with no overall effect (e.g., outline overlap, offset of the skirt from the raft and of the structure to the skirt, and many others). The parameters were initially changed in isolation and then combined with other amended parameters, until finally a successful honeycomb scaffold was obtained (Figure 8). Although the scaffold was not porous in the *xz* plane with only vertical channels present, it provided the necessary groundwork to advance the construct.

The process was characterized by iterative, trial-and-error experimentation, where each parameter adjustment informed subsequent modifications. In this discussion, the parameters are analyzed in the format appearing on the Simplify3D slicing software, which lists the tab headings as “layer”, “infill”, “additions”, “temperature”, and “Other” (Table 3). The most influential parameters, as outlined in Table 3, are discussed respectively in detail below.

### 3.1 Adjusting the Parameters

#### 3.1.1 Top, Bottom, Side Layers

Originally, these layers provided a solid outer shell to form a 20% infill structure. As explained previously, the original structure required a solid surface, so the original parameters included multiple shells on the superior and inferior sides and outer perimeter of the construct. Since the required result was a porous scaffold with empty spaces throughout, these layers were immediately omitted from the parameters to prevent a solid superior, inferior, and/or perimeter to the disc.

#### 3.1.2 Infill Pattern

This parameter was key in later designs. This parameter complements the “infill percentage” parameter as such. Originally, 20% infill was set to reduce the amount of material used. However, this infill density obstructed accurate fabrication of the designed pores. For the scaffolds, this ‘Infill Pattern’ parameter was initially adjusted to 100%, with the premise that it should result in the printer fabricating the design in its entirety, including pores. However, the prints still had to be stopped prematurely because of potential damage to the nozzle, meaning that at this stage, all the prints were failed misprints even with adjusting minor parameters (Figure 6). However, the “infill pattern” was ultimately the key parameter which created the first successful scaffold.

**Figure 6:**
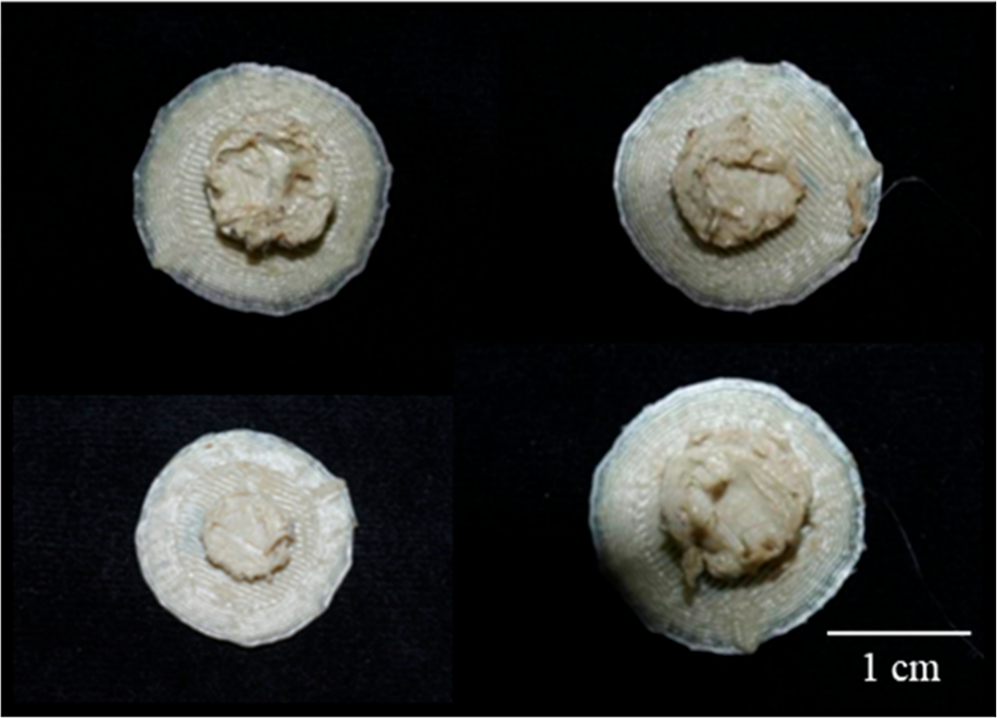
A series of scaffold misprints during the optimization process.

The results above prompted the exploration of additional parameters, such as the infill pattern. Specifically, two patterns were tested: rectilinear and honeycomb (Figure 7). This was because only these two patterns would allow alternating angles coupled to an adjusted infill percentage to create a porous infill. In fact, when coupled with the infill percentage and angle offsets (Result and Discussion in subsequent sections), the selected infill pattern successfully produced a porous structure, marking the first successful outcome.

**Figure 7:**
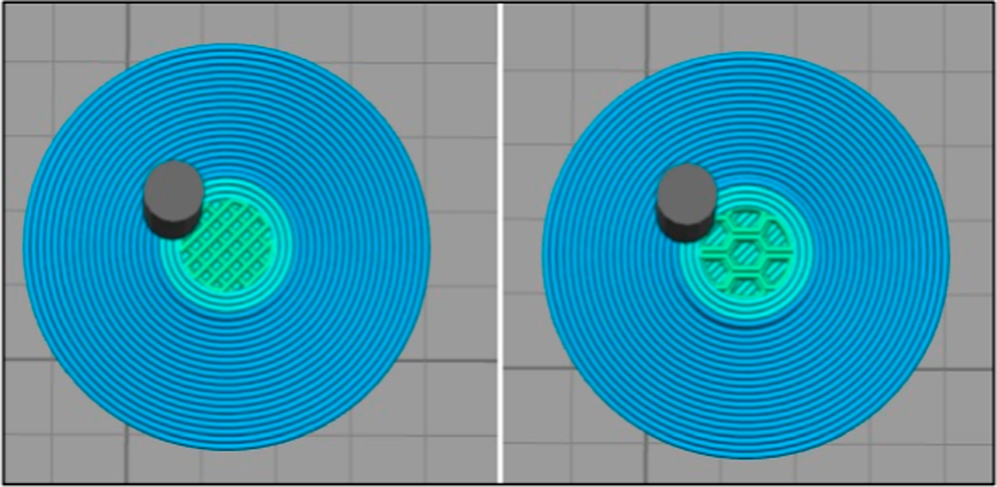
3D software interface showing rectilinear (left) and honeycomb (right) infill patterns (Version 4.1.2).

When the infill percentage was set to below 100%, these two infill patterns; namely honeycomb and rectilinear created a porous scaffold. This was because the software then designed the construct using the spaces/voids from the stipulated infill percentage and combined them with these specific printing patterns at controlled alternating printing angles (explained in the coming sections) to create a final construct with controllable geometry and porosity.

Previous studies on polylactic acid (PLA) have shown that the rectilinear printing pattern at alternating horizontal–vertical has a higher impact resistance than a triangular printing pattern, attributable to the transversal geometry which would essentially absorb more energy during crack propagation.^53^ This study also showed that when relating the sample mass to the impact resistance, the rectilinear printing pattern proved the most efficient out of the printing patterns tested (rectilinear, grid, triangle, and honeycomb). The printing pattern is therefore a key parameter in the mechanical behavior of a scaffold, where the rectilinear printing pattern has been proven to be the strongest.^54^

#### 3.1.3 Infill Angle Offsets

This parameter also played a crucial role in later design stages. The movement angle of each layer can be customized for the specific print. By default, it is set to 0°, meaning the nozzle moves in a straight, parallel path along the *x*-axis and then reverses direction in a similar manner, assuming a rectilinear infill pattern is used. The infill angle offset can be a complex and sometimes ineffective setting, as it works in tandem with the chosen infill pattern. If the selected pattern has a predefined angle offset such as in honeycomb, grid, or triangular patterns, the infill angle offset typically has a minimal or unpredictable impact on the final print structure.

When the infill angle offset was exploited in combination with a rectilinear printing pattern, the final construct was modifiable as a scaffold. In fact, the optimized scaffold was ultimately designed as a solid cylinder in the Autodesk Inventor, with the same dimensions as before (i.e., 10 mm by 4 mm) but sliced differently. The infill pattern and infill angle offset as well as infill density/percentage combined (described in Sections 3.1.2, 3.1.3, and 3.1.4) provided a 50% porous scaffold with a regular geometry and an interconnected network of pores throughout.

#### 3.1.4 Infill Percentage

This is essentially the density of the construct; 90% infill percentage results in a structure that is theoretically 90% of its original design in terms of weight and volume, albeit with unchanged outer dimensions. The other 10% is empty space homogeneously distributed throughout the structure due to the rigid restrictions of the slicing software. Similarly, a 10% infill percentage would result in a structure that is only 10% in weight and volume, with 90% of the construct being empty space. The size and distribution of the space are predominantly determined by the infill pattern and angle offsets as described previously, as exemplified below (Figure 8).

**Figure 8:**
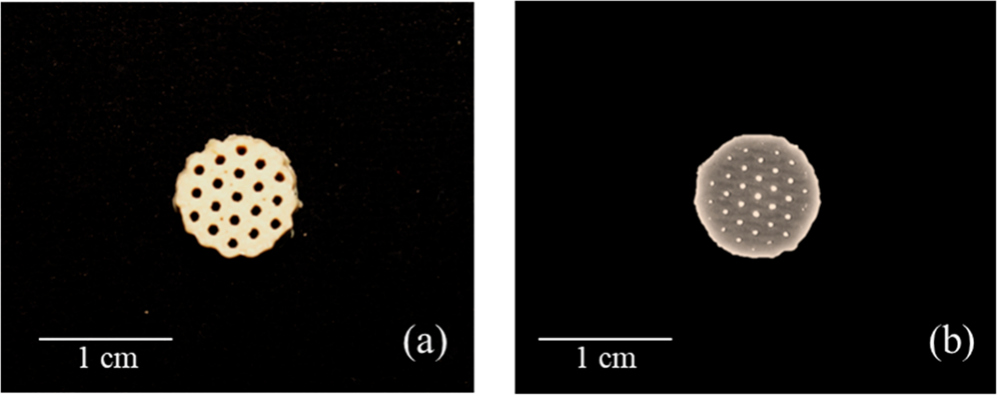
Both structures (a,b) were printed with a honeycomb infill pattern, albeit at different densities. Structure (a) was printed at 60% infill and Structure (b) was printed at 80% infill. The difference in color is due to a light source placed under Structure (b) to determine the presence of pores, almost invisible to the naked eye.

As reported in the literature,^55^ the trabecular bone has a porosity of 50%–90%. To maintain desired pore sizes for a scaffold with this intention, 50% porosity was chosen for this project, resulting in equal space and strut fill. To confirm its optimality, 40% and 60% infill discs, identical to the 50% porous disc, were printed and imaged (Figure 9 and Figure 10, respectively).

**Figure 9:**
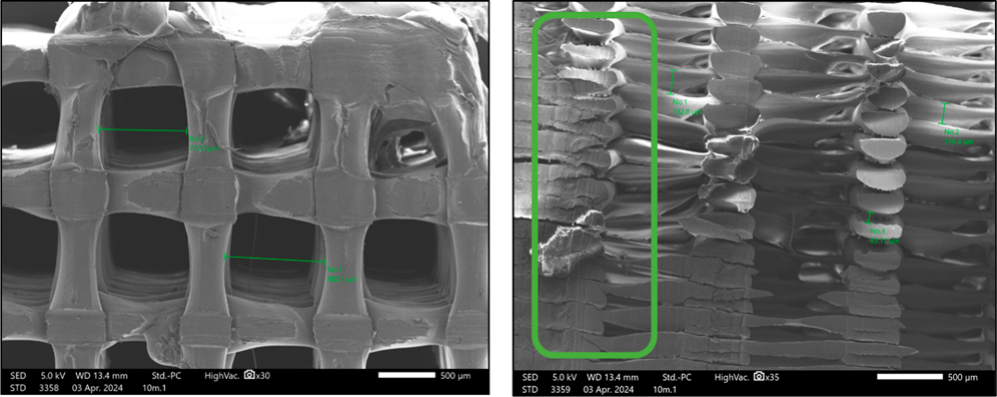
A PEEK scaffold with 60% porosity (and rectilinear print pattern). The *xz* pore width was ∼800 μm, the *xy* pore height ∼169 μm, and the *xz* pore height ∼80 μm. However, the print was uneven and the sides were compressed (denoted in green).

**Figure 10:**
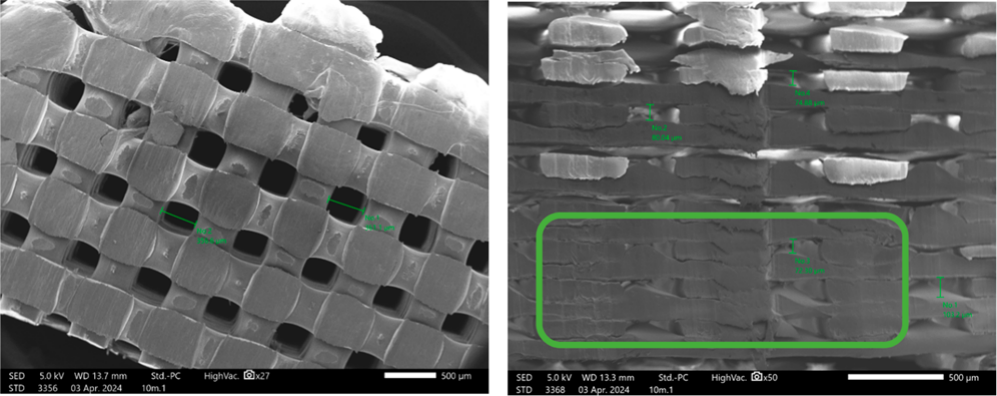
A PEEK scaffold with 40% porosity (and rectilinear print pattern). The *xz* pore width was ∼358 μm, the *xy* pore height was ∼78 μm, and the *xz* pores were virtually nonexistent. The print was adequately even, but all the layers were compressed, leaving no room for empty spaces/pores, with the more grievous section denoted in green.

The optimal 50% disc, henceforth referred to just as disc, had a basic printing structure (using the parameters in Table 3) which is later referred to as the “cell” when part of a cellular structure. This basic unit cell is very symmetrical and easily replicable (Figure 11a), and it is very similar to the “diamond unit cell” reported in the literature.^45^ A unit cell which looks like this would create a scaffold with an open-cell structure, where the repeating units are interconnected—allowing communication and flow throughout the scaffold.^56^

**Figure 11:**
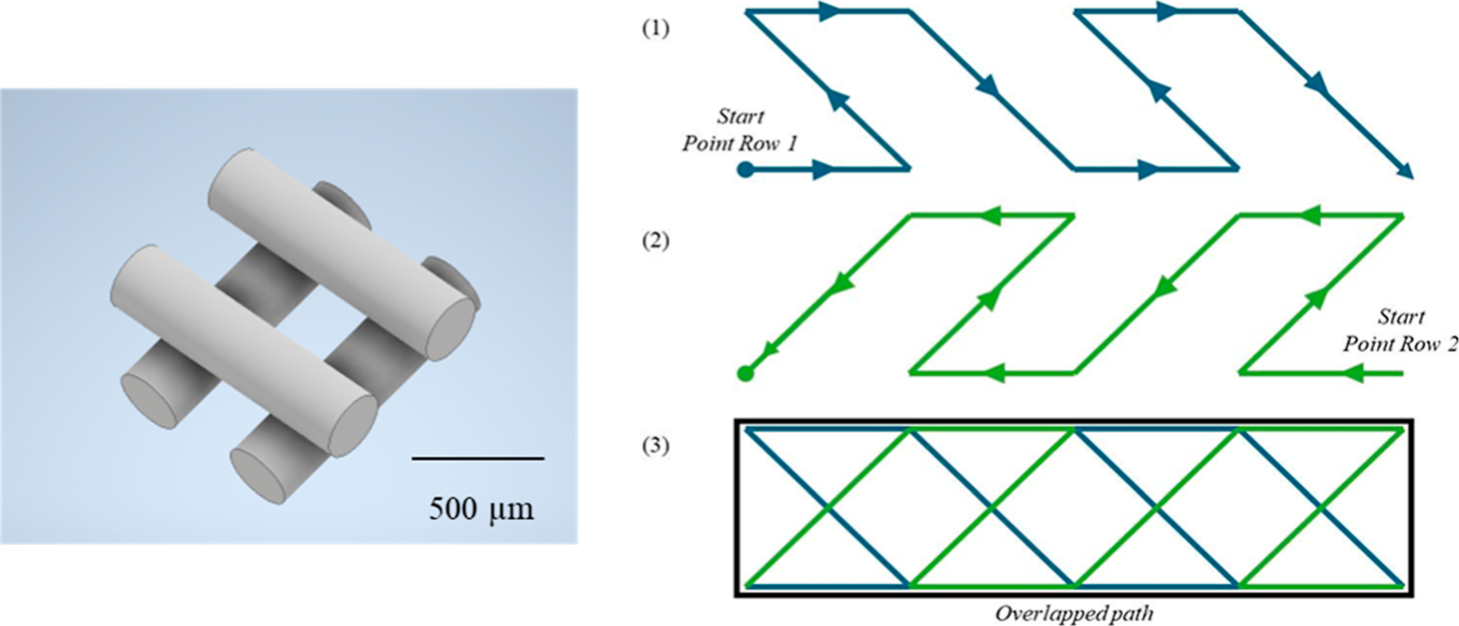
(a) The basic unit cell of the optimized scaffolds. The spaces between the struts are equal to the thickness of the struts themselves, going both horizontally and vertically. The viewing angle is not by coincidence—the optimized infill angle offsets were set to be as (b) + 45° and −45° alternating printing angles with every 0.1 mm thickness layer. The alternating linear struts were stacked perpendicularly to create a 10 mm by 4 mm scaffold construct.

These unidirectional linear layers were essentially stacked in alternating rows to create a regular, perpendicular pattern (Figure 11b). At 50% infill, this cellular unit also predicted an interconnected network of pores, which was proven by using SEM (Figure 16).

It is worth noting that this “diamond” architecture aligns with what is considered “the most widely applied tissue-like structure”,^50^ with similar work conducted by other researchers.^57^ These studies additively manufactured their polycaprolactone/nanohydroxyapatite scaffolds at 0 °C/90° layers, while this present study printed the scaffolds at +45 °C/–45° layers, essentially giving rise to the same result.

#### 3.1.5 Outline Overlap

This determines the overlap between the infill itself and the outer perimeters, and by extension, the amount of material at the edges. Since no perimeter shells were used, this was set at 50%. The software did not allow for a 100% outline overlap, and when lower values were tried, the edges were rough and disorganized; some of the structures had to be stopped midprinting. The perimeter of the optimized scaffold was almost solid—in fact, no pores were seen with the naked eye on the sides of the final disc (Figure 12). Despite this, 50% was determined to give the best results, as the structure was printed to completion without any damaging effect on the otherwise interconnected pore network.

**Figure 12:**
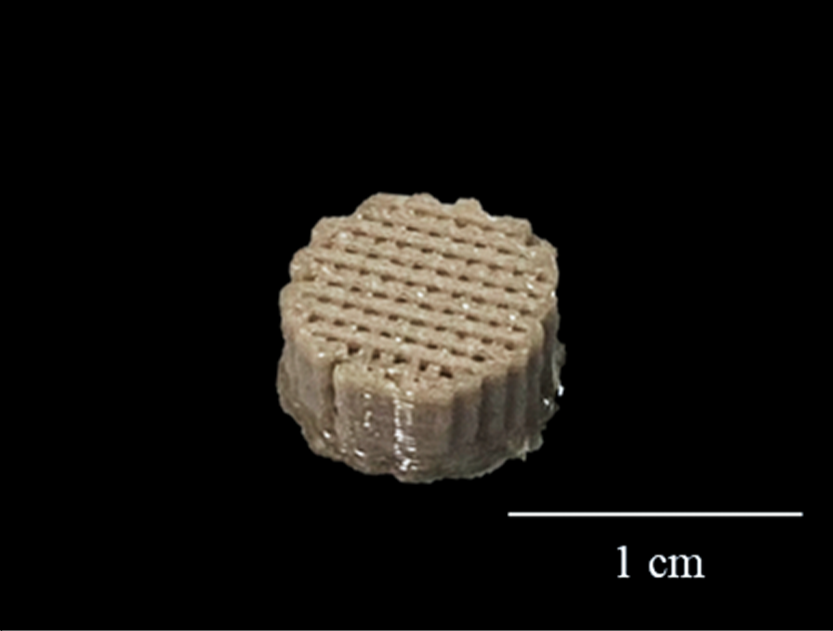
Final optimized scaffold. Note the absence of pores on the side of the disc, but the homogeneous, regular, structured pores at the top.

#### 3.1.6 Skirt/Brim Outlines

The skirt and brim outlines were crucial in the printing process. This parameter essentially affects the width of the skirt, which is the excess area laid down before the actual structure starts to be printed (Figure 13). This helps the printed construct to adhere better and thus mitigate the risk of any misprints. When 10 outlines were selected, the print lifted off the bed, and so, the number of outlines had to be increased. When this number was set at 20, this led to satisfactory adhesion of the print to the bed, and so, “20 outlines” were the retained parameter value.

**Figure 13:**
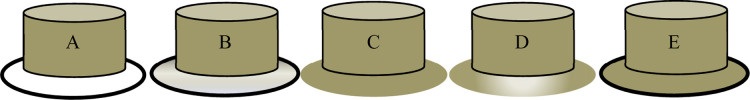
Preset additions in the slicing software. (A) Skirt (black outline), (B) Brim (faded), (C) Raft (solid), (E) Raft with brim, (F) Raft with skirt.

#### 3.1.7 Skirt Offset from the Part

Since the skirt is separate from the construct, there is an option as to “how separated” the layers of the skirt are printed in relation to the footprint of the construct. The role of the skirt is to prime the nozzle head, but used correctly; it can also aid in anchoring the edges of the print. Therefore, an offset of 0 was chosen, so the outlines of the skirt were approximated to the base layer of the construct.

#### 3.1.8 Printing Temperature

The printing temperature also required optimization. The original solid prints, which had been used in a previous project and were thus successful, were being printed at 486 °C. When this temperature was tested on an early designed construct, the extruded material was charred in places, proving that the printing temperature was too high. There is no consensus about the optimal printing temperature of PEEK. However, this would be contingent on a nonburnt print which does not conglomerate in a disorganized mass due to high printing temperatures and prints accurately without any missing struts due to low printing temperatures. Different temperatures were tested to fulfill these requirements, ranging from 520 °C albeit for a different shape, all the way down to 435 °C. This latter temperature was found to be optimal for consistent, repeatable results with no charred parts.

#### 3.1.9 Heated Bed Temperature

An insufficiently heated bed can cause premature cooling of the primary layers, leading to poor attachment of the construct to the bed; however, a bed maintained at a high temperature has its own drawbacks. When using the bed at the original 150 °C, although a neat, successful scaffold was created with clear channels, it exhibited the “elephant’s foot” issue,^58^ where lower layers compressed and flattened toward the bottom. This was likely due to an excessively hot bed, which was resolved by lowering the temperature from 150 to 130 °C (Figure 14).

**Figure 14:**
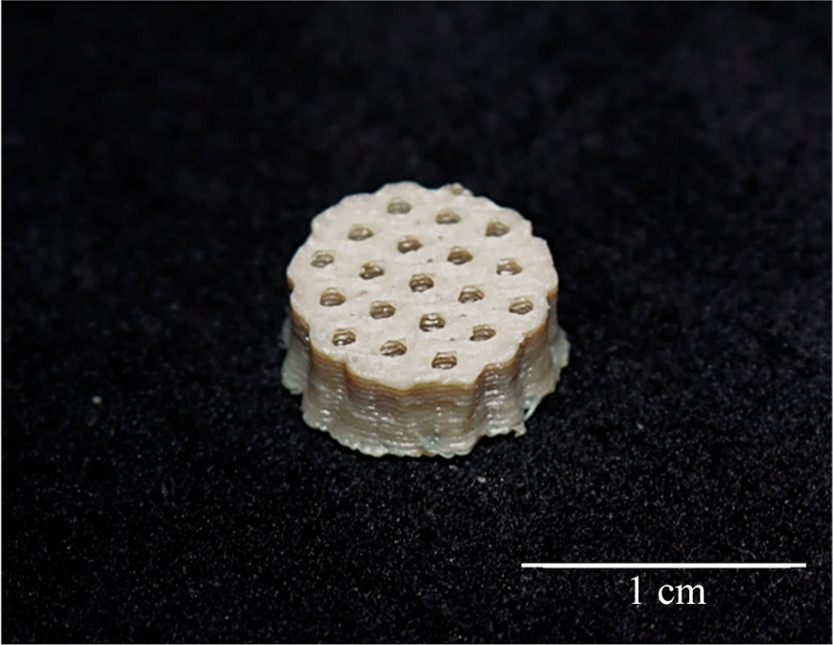
Elephant’s foot issue on an early prototype (honeycomb) of the scaffold.

#### 3.1.10 Cooling Layer 1

The print’s attachment to the bed was unpredictable at first, and this can be due to a temperature difference which causes the first layer to cool down too quickly without attaching itself to the bed. Therefore, this setting on the printer lets the user adjust the cooling layer of the first layer, because the attachment of the printed part essentially depends on the attachment of that first layer. This was 0% (fan strength) to start with, but after several adjustments as the print was still unpredictably attaching to the bed, a second setting was added. Layer 2 was set for the fan speed at 100%, with the option “Blip fan to full”. This was to allow the first layer of the part to adhere to the bed but prevent any compression of “unset” layers which could potentially lead to the elephant’s foot issue (Figure 14).

#### 3.1.11 Extrusion Multiplier

This setting allows for adjustment of the amount of material coming out of the nozzle. It is a range that goes up all the way to 1 (equivalent to 100%). The default setting for the printer was at 0.9; however, with the early prototypes of the scaffold, it was evident that there was too much material being extruded, so this value was modified to 0.85 and later to 0.8. However, once the other parameters were confirmed and a scaffold was satisfactorily printed, this was reverted to 0.9 because the 0.8 extrusion multiplier was giving rise to misprints.

#### 3.1.12 Printing Speed

This setting is usually a fine balance between slowing the print sufficiently to obtain all the desired detail but not to the point where it results in unnecessary material extruding in any given spot due to the nozzle lingering. It also needs to be fast enough to “bridge” the empty spaces in the scaffold without the strut sagging, but it cannot be too fast as there will be a misprint when the printer tries to coordinate the extrusion multiplier and printing the desired shape. This step was particularly challenging, as the literature-reported values did not yield satisfactory results for Apium P155. The default setting of 4800 mm/min was progressively reduced incrementally to 800 mm/min, which ultimately produced the desired outcome.

#### 3.1.13 Generate Support Material

The printer encountered difficulty with the circular scaffold geometry, resulting in excessive material deposition. This was contrary to the desired outcome, and the extrusion multiplier was initially suspected as the cause (as explained above). While this might have been the case, once the “Generate Support Material” setting was switched off, there was a significant improvement in the result.

#### 3.1.14 Internal Thin Wall Type

This setting, when selected for “Allow single extrusion fill”, determines how internal thin wall gaps should be filled within the model. It essentially allows any extremely thin parts to be printed in a single extrusion fill motion (Figure 15a), rather than the normal back and forth motion of the printer (Figure 15b). This is helpful for thin sections, and it was a crucial turning point in the optimization process of the scaffold as this setting allowed all the internal struts to be printed as intended, without any disordered, excess material in lieu of the pores.

**Figure 15:**
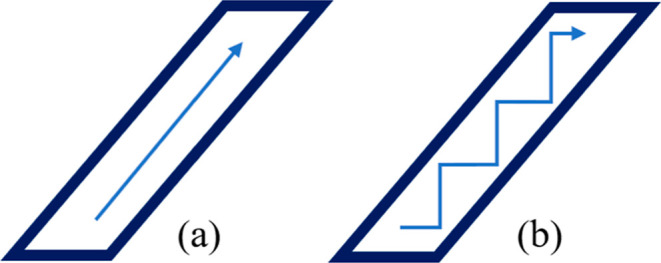
Illustration of single-extrusion fill strategies. (a) shows a fill path aligned parallel to the main design direction. (b) shows a rectilinear pattern that does not automatically follow the intended design orientation. When the option ‘Allow single extrusion fill is selected within the slicing software’, the printer selects the optimal path based on the geometry, rather than defaulting to the pre-defined pattern.

These parameters were not the only ones which were adjusted but rather the ones which had the most influence on the overall construct. Several studies have attempted PEEK scaffolds before with a variety of internal structures, including trabecular geometry,^59^ even very similar geometry to this current study.^60,61^ However, none of the studies gave an exacting and repeatable protocol as to the fabrication of these scaffolds in FDM PEEK especially when using a nonheated printing chamber.

The fabrication of an open-pore scaffold marked a significant advancement in this research. While a solid disc had already been successfully produced, achieving a porous structure proved significantly more challenging. Over 80 printing parameters were systematically explored and iteratively modified, initially in isolation and later in combination, to attain the desired scaffold architecture. The final optimized scaffold was observed under SEM and confirmed to have an interconnected network of adequately sized pores ranging from 100 to ∼400 μm­(Figure 16).

**Figure 16:**
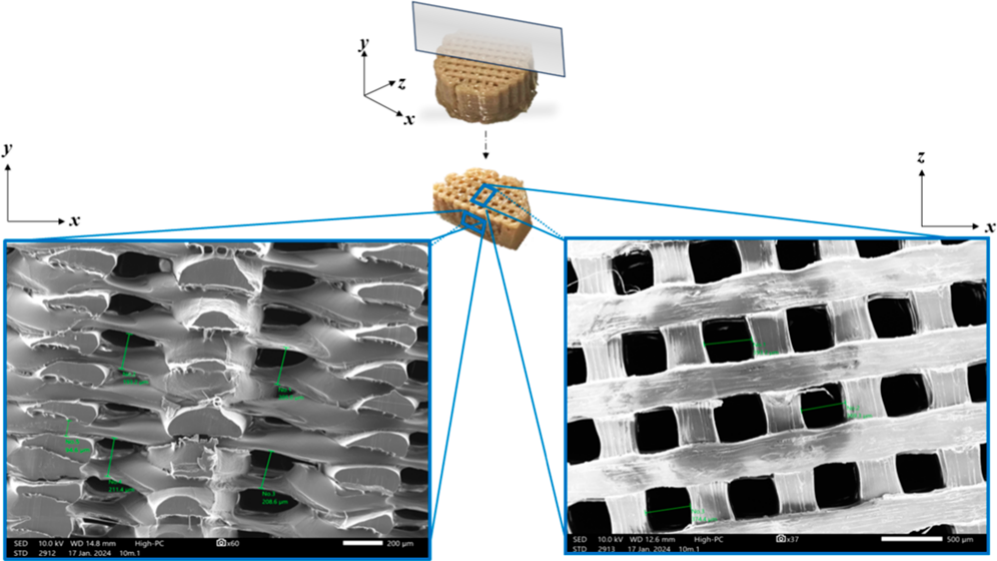
Final scaffold at the top, with the blade angle as demonstrated. The resulting half of the disc was imaged from two directions—the *xy* direction (left) and the *xz* direction (right). The pores were confirmed to be ∼100 μm in depth (*xy* direction) and ∼4000 μm in width (*xz* direction).

### 3.2 Preliminary Biological Work

Once the fabrication of a porous scaffold with ideal pore dimensions was fabricated, a cell culture model was used to assess the biocompatibility of the construct, both in its surface chemistry and topology, as well as its porous geometry. A primary human osteoblast cell model was used to assess the biological potential of the PEEK scaffolds with cells microseeded and cultured over a period of 21 days. SEM images of the internal surfaces of the porous scaffolds show that the statically seeded human osteoblasts had attached throughout the internal structure of the fabricated scaffold (Figure 17).

**Figure 17:**
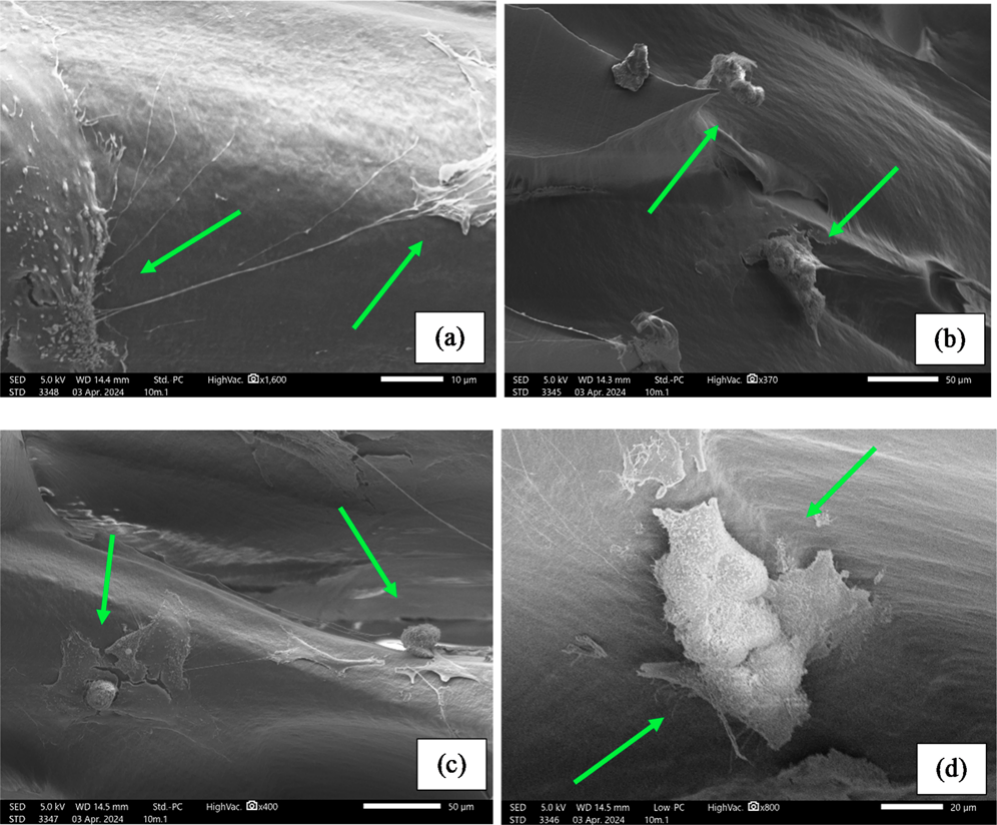
Preliminary cell work on the fabricated scaffolds, with the green arrows marking the attached human osteoblasts to the PEEK substrate.

This meant that the scaffold is biologically able to host human osteoblasts, giving rise to successful adhesion, which is the first step of successful osseointegration.^62–66^ Figure 17d also shows the presence of what appears to be an extracellular matrix, suggesting the initiation of mineralization or, at least, matrix synthesis. This means that the human osteoblasts have actively integrated with the fabricated PEEK scaffold and are on the trajectory to osteoid matrix deposition and, by extension, osseointegration *in vivo*. Further biological work would be needed to confirm and identify any matrices produced by these human osteoblasts when within the fabricated PEEK scaffold.

By optimizing a combination of the aforementioned parameters, a successful scaffold was fabricated. The key parameters were the infill density, paired up with an appropriate printing pattern, whilst alternating the printing angle offsets with every layer, whichessentially creates a repeatable and uniform porous geometry. This is an outcome that is typically difficult to achieve yet crucial for promoting enhanced cellular response and soft tissue integration.^67^ While porous scaffolds for bone tissue engineering have been successfully fabricated in previous studies,^3,57,60,68–70^ these efforts predominantly utilized alternative materials such as polycaprolactone (PCL), composite systems, or employed entirely different fabrication strategies. Notably, Elhattab et al. (2019) presented scaffolds visually similar to those produced in this study; however, the specific parameters required for reproducible fabrication were not disclosed.

These parameters were then translated to fabricate a variety of geometries. Through the maintaining of these slicing parameters and exploring different infill patterns, the possible designs for reproducible scaffolds expand almost infinitely, unlocking endless architectural possibilities tailored to diverse clinical demands. To prove this, the author fabricated two other scaffolds in a “wiggle” infill pattern (Figure 18a) and a different one in a “fast honeycomb infill pattern” (Figure 18b). Their successful fabrication proves that the parameters established throughout this methodology are repeatable, reproducible, and able to fabricate any desired scaffold in PEEK using a filament printer.

**Figure 18:**
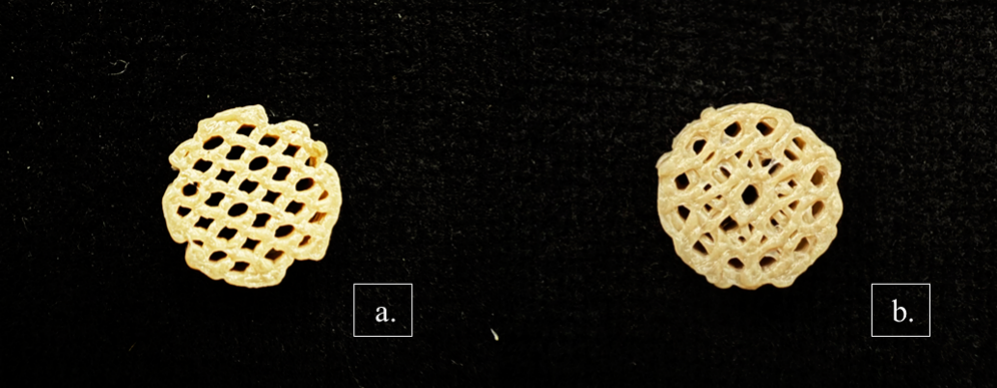
(a) Scaffold of 10 mm by 4 mm in “wiggle” infill print at 50% infill and alternating layers +45, −45, (b) Scaffold of 10 mm by 4 mm in “fast honeycomb” infill print at 50% infill and alternating layers +45, −45.

To the best of the author’s knowledge, this study is one of the first to describe a parameter-optimised protocol for fabricating 100% PEEK scaffolds without requiring a heated chamber to control warpage; instead its effects are achieved through a precise balance of cooling, bed temperature, nozzle temperature, and printing speed. The majority of available studies also employ computer-aided design (CAD) to generate porous geometries. In contrast, this present study relies solely on the slicing parameters, with the final scaffolds using the RL printing pattern at +45°, –45°, and 50% printing infill to create a porous structure. These scaffolds met physical and mechanical requirements for bone tissue engineering, complying with ISO 178 ^71^ as well as satisfactory biocompatibility as outlined in this study. The parameters used to fabricate these FDM scaffolds have been detailed to enable a straightforward reproduction of the constructs. The novelty of this study lies in the disclosed, generalizable slicing parameters, which can be applied to various patterns and potentially to multiple materials using FDM.

## 4 Conclusion

In this study, conventional computer-aided design (CAD)-based scaffold design approaches proved inadequate for achieving the required printability and structural fidelity. To address this, we adopted a novel strategy centered on the direct manipulation of slicing software parameters to generate the desired architecture. Through iterative optimization, we achieved reproducible fabrication of structurally consistent porous scaffolds, with pore dimensions falling within the optimal range for osteoblast infiltration.

This approach is readily translatable across PEEK filament printing, relying on the outlined parameters and the targeted adjustment of the infill pattern and density to produce a range of porous, consistent, and tailorable scaffolds. The protocol should, in principle, also be applicable to filled PEEK formulations (incorporating additional constituents), provided that filament-based extrusion is used. Further investigation is warranted to assess its applicability to both filled PEEK and alternative alloplastic materials such as polycaprolactone (PCL) and polylactic acid (PLA), with an emphasis on slicing parameter optimization rather than CAD-based design modifications.

Overall, this optimization strategy demonstrates that precise control over slicing software parameters can effectively overcome design limitations, enabling the successful production of PEEK scaffolds tailored for bone tissue engineering applications.
